# Preventing traumatic childbirth experiences: 2192 women’s perceptions and views

**DOI:** 10.1007/s00737-017-0729-6

**Published:** 2017-05-29

**Authors:** M. H. Hollander, E. van Hastenberg, J. van Dillen, M. G. van Pampus, E. de Miranda, C. A. I. Stramrood

**Affiliations:** 1 Department of Obstetrics, Radboud University Medical Center, Geert Grooteplein-Zuid 10, 6525 GA Nijmegen, The Netherlands; 2 Department of Obstetrics and Gynecology, OLVG Oost Hospital, Amsterdam, The Netherlands; 3 Department of Obstetrics, Academic Medical Center, Amsterdam, The Netherlands; 4 Department of Obstetrics and Gynecology, University Medical Center, Utrecht, The Netherlands

**Keywords:** Traumatic, PTSD, Prevention, Postpartum, Childbirth, Communication, Control, Interventions, Support

## Abstract

The purpose of this study is to explore and quantify perceptions and experiences of women with a traumatic childbirth experience in order to identify areas for prevention and to help midwives and obstetricians improve woman-centered care. A retrospective survey was conducted online among 2192 women with a self-reported traumatic childbirth experience. Women were recruited in March 2016 through social media, including specific parent support groups. They filled out a 35-item questionnaire of which the most important items were (1) self-reported attributions of the trauma and how they believe the traumatic experience could have been prevented (2) by the caregivers or (3) by themselves. The responses most frequently given were (1) *Lack and/or loss of control* (54.6%), *Fear for baby’s health/life* (49.9%), and *High intensity of pain/physical discomfort* (47.4%); (2) *Communicate/explain* (39.1%), *Listen to me* (*more*) (36.9%), and *Support me* (*more/better*) *emotionally/practically* (29.8%); and (3) *Nothing* (37.0%), *Ask for* (26.9%), or *Refuse* (16.5%) *certain interventions*. Primiparous participants chose *High intensity of pain/physical discomfort*, *Long duration of delivery*, and *Discrepancy between expectations and reality* more often and *Fear for own health/life*, *A bad outcome*, and *Delivery went too fast* less often than multiparous participants. Women attribute their traumatic childbirth experience primarily to lack and/or loss of control, issues of communication, and practical/emotional support. They believe that in many cases, their trauma could have been reduced or prevented by better communication and support by their caregiver or if they themselves had asked for or refused interventions.

## Introduction

Giving birth can be a traumatic experience for women. The extreme outcome of a traumatic birth experience, posttraumatic stress disorder (PTSD), has been an increasingly popular topic of research (Ayers 2004; Grekin and O’Hara 2014; Ayers et al. 2016; Soet et al. 2003; O’Donovan et al. 2014; Stramrood et al. 2011; Rijnders et al. 2008; Elmir et al. 2010; Harris and Ayers 2012). A recent meta-analysis of 78 studies found the prevalence of postpartum PTSD due to childbirth to be 2.9% in community samples (Grekin and O’Hara 2014). A recent systematic review on risk factors for PTSD following childbirth, divided these into prebirth, during birth, and postpartum risk factors (Ayers et al. 2016). The strongest prebirth factors were depression in pregnancy, fear of childbirth, poor health or complications in pregnancy, and a history of PTSD. Risk factors during birth were negative subjective birth experiences, having an operative birth (unplanned cesarean section or operative vaginal delivery), lack of support, and dissociation. Postbirth risk factors associated with PTSD were poor coping, stress, and depression. Prospective studies into predictors of “finding birth traumatic” found similar factors (Soet et al. 2003; O’Donovan et al. 2014). Researchers have thus far mainly focused on women who developed postpartum PTSD, while they are part of a much larger group of women (9.1–45.5%) who experienced their delivery as traumatic (O’Donovan et al. 2014; Stramrood et al. 2011; American Psychiatric Association 2000).

A Dutch study investigating recall of birth experience 3 years postpartum found that 16.3% of low-risk women looked back negatively on their delivery, especially in case of referral from primary to secondary care (Rijnders et al. 2008).

Why do so many women experience birth as traumatic? A meta-ethnography of ten qualitative studies for which women had been interviewed about their traumatic birth experiences found as most important themes topics concerning communication, being seen as a person and taken seriously, and emotional support during labor (Elmir et al. 2010).

Quantitative measures on attributions and prevention of traumatic delivery experiences could supplement and extend current knowledge, which is thus far based on qualitative studies of perceptions and experiences and quantitative studies of risk factors. Therefore, the objectives of this study were to identify (1) women’s attributions of their trauma, (2) what they feel their caregivers could have done differently, (3) what they themselves could have done to prevent the trauma, and (4) differences between primi- and multiparous women.

## Methods

### Setting/research design

In the Netherlands, the maternity care system is divided into primary care and secondary care. Low-risk women receive care from independent community midwives during pregnancy and delivery (primary care). High-risk women (or those who become so) are cared for in an obstetrician-led hospital setting (secondary care). Referral from primary to secondary care takes place when complications arise at any point or if an increased risk of complications is anticipated, as indicated by national guidelines (CVZ 2003).

For this study, permission was sought from and waived by the medical ethics committee of the Radboud University Nijmegen.

A retrospective survey was conducted among women with one or more self-reported traumatic birth experience. Data were collected in March 2016.

### Participants

Women were eligible for participation if they were at least 18 years old, able to complete a Dutch questionnaire, and if their traumatic childbirth experience occurred in or after 2005.

### Materials

The questionnaire was made available via Survey Monkey. Questions were based on themes and risk factors identified in the literature (Grekin and O’Hara 2014; Ayers et al. 2016; Soet et al. 2003; O’Donovan et al. 2014; Stramrood et al. 2011; Rijnders et al. 2008; Elmir et al. 2010; Harris and Ayers 2012), with emphasis on the findings by Ayers et al. (2016) and Elmir et al. (2010). The questionnaire was designed specifically for the purpose of this study and contained 35 items in total. In addition to items concerning medical details and basic demographic characteristics of the participants, the three main questions concerned the participants’ attribution of their trauma, what they thought their caregivers could have done to prevent the trauma, and what, if anything, they themselves had wanted to do differently. Given options ended with the option *other*. Participants were invited to explain their responses in a free text field. For questions (2) and (3), a maximum of three possible responses was set and participants had to rank their chosen answers, numbering them 1–3. It also contained questions about postpartum follow-up as well as validated questionnaires on posttraumatic stress (PCL-5), coping (sense of coherence), and social support (Oslo social Support Scale, OSS-3). The results of these questionnaires are the subject of a second paper from this study.

To improve the quality of the questionnaire, it was reviewed by members of the CAPTURE group (Childbirth and Psychotrauma Research Group), the Committee for Patient Communication of the Dutch Association of Obstetrics & Gynaecology (*Commissie Patiëntencommunicatie NVOG*), and the committee currently designing a Dutch national guideline on PTSD following childbirth and traumatic birth experiences. Furthermore, it was pilot-tested by two women who had a traumatic birth experience themselves to identify potential problems with Survey Monkey, unclear instructions, or other content issues.

### Procedures

The invitation to participate in the study read: “Did you have a traumatic birth experience? If so, please fill out this questionnaire.” The question was framed this way on purpose, so it would be clear to the participants that the subject of the study was emotional trauma, not physical. In addition, in the Netherlands, the word trauma is generally understood by lay people as psychological trauma. There were no selection criteria about the nature of the emotional trauma or its intensity. If the participants deemed their experience traumatic, they were eligible for inclusion.

Participants were recruited through online invitations posted on a website created for the purpose of this study (www.traumatischebevalling.nl), a Facebook page (www.facebook.com/traumatischebevalling), and a Twitter account (@BevallingTrauma). Midwives and gynecologists in the authors’ own networks were approached and requested to share the invitations on social media. Furthermore, the invitation was frequently shared by various other professionals (such as women’s coaches, EMDR (eye movement desensitization and reprocessing) therapists, psychiatrists, psychologists, and lactation consultants) and by lay people, as well as by the Royal Dutch Organisation of Midwives (KNOV) and the Netherlands Association of Obstetrics and Gynaecology (NVOG). In total, the first Facebook invitation on the page created for this study was shared 243 times and reached 28,510 Facebook users. The invitation was, at our request, also posted on pages of several Dutch support groups for pregnancy and childbirth such as the Birth Movement (*Geboortebeweging*), Traumatic Childbirth and Postpartum Depression group (*Traumatische Bevalling & Postnatale Depressie*), HELLP Syndrome Foundation (*HELLP Stichting*), and Association for Parents of Incubator Babies (*Couveuseouders*).

The filled-out questionnaires were imported from Survey Monkey into the SPSS version 22 (IBM Corporation Inc., Armonk, NY, USA). Descriptive statistics were used to summarize the characteristics and opinions of the study subjects. Chi-square tests were used to examine associations between patient’s characteristics and their attributions of the trauma. Difference between multiple groups was compared using analyses of variance (ANOVA), with the Games-Howell test being used as posthoc analysis. *p* values of less than or equal to 0.05 were considered significant. Women’s responses in regard to frequency and ranking were compared for parity (primipara/multipara), level of care during delivery (primary care/secondary care/referral), and educational methods used to prepare for delivery. They were also compared to overall statistics in the Dutch National Perinatal Registry (Brouwers 2014). This was done in order to (1) compare sample characteristics to population characteristics to determine if a representative sample had been obtained and (2) evaluate whether previously known risk factors for PTSD following childbirth (e.g., preterm birth, instrumental delivery, emergency cesarean section) were indeed more prevalent in the current sample than in the general population.

## Results

The total number of questionnaires started was 2634. After removing doubles (based on IP address), participants who had given birth before 2005, participants with impossible answers (e.g., cesarean section at home), and participants who quit the questionnaire before the main question on the cause of the traumatic experience, 2192 responses remained for analysis. The characteristics of the participants are shown in Table 1. Comparison with maternal characteristics from the Dutch Perinatal Registry is shown in the last column (Brouwers 2014). The study population differs significantly in ethnicity, parity, gestational age at delivery, mode of delivery, and level of care during pregnancy and delivery compared to the general population.

**Table 1:** Characteristics of the participants (*n* = 2192) compared to the reference group

Characteristic	Participants *n* (%) or mean{SD}	95% Confidence interval	Dutch perinatal registry^a^
Age (*n* = 2192)			
At time of survey	33.1 {5.5}		
At time of (traumatic) birth	29.6 {4.4}^*^		31.0 yr {4.9}
Gestational age at time of traumatic childbirth			
16–32 weeks	133 (6.1)		>22 wks: 1.5^b^
32–37 weeks	243 (11.1)^*^	9.8–12.4	6.1
37–42 weeks	1688 (77.0)^*^	75.2–78.8	89.8
>42 weeks	127 (5.8)^*^	4.8–6.8	1.3
Unknown	N/A		1.3
Years since traumatic childbirth			
<2 years	999 (45.6)		
2–5 years	684 (31.2)		
5–12 years	509 (23.2)		
Ethnicity (*n* = 2192)			
Dutch	2004 (91.4)^*^	90.2–92.6	74.3
Not Dutch	188 (8.6)^*^	7.4–9.8	25.7
Parity at time of survey (*n* = 2178)			
Mean parity	1.66 {0.8}^*^		1.71^c^
Parity at time of traumatic birth experience (*n* = 2178)			
Primiparous	1737 (79.8)^*^	78.1–81.5	45.2
Multiparous	441 (20.2)^*^	18.5–21.9	54.8
Responsible caregiver during pregnancy (*n* = 2180)			
Midwife	1146 (52.6)	50.5–54.7	50.7
Obstetrician	345 (15.8)	14.3–17.3	14.6
Both (referral from primary to secondary care during pregnancy)	677 (31.1)^*^	29.2–33.0	34.7
Mode of delivery (*n* = 2176)			
Spontaneous vaginal delivery	919 (42.2)^*^	40.1–44.3	74.9
Operative vaginal delivery	577 (26.5)^*^	24.6–28.4	8.7
Secondary cesarean section	627 (28.8)^*^	26.9–30.7	8.6
Primary cesarean section	53 (2.4)^*^	1.8–3.0	7.8
Responsible caregiver during delivery (*n* = 2147)			
Midwife (primary care)	122 (5.7)^*^	4.7–6.7	27.4
Obstetrician-led (secondary care)	1098 (51.1)	49.0–53.2	49.4
Both^d^ (referral during labor or directly postpartum)	927 (43.2)^*^	41.1–45.3	23.2

^*^Significantly different from Dutch perinatal registry, *p* ≤ 0.05

^a^Source, unless otherwise specified: Dutch Perinatal Registry (Brouwers 2014)

^b^Data collection starts at 22 weeks gestation, so no data between 16 and 22 weeks are known

^c^“Mean parity” (CBS 2014)

^d^Referral from primary to secondary care

### Attribution of trauma

Most frequently perceived causes of or contributions to the traumatic experience, were *Lack and/or loss of control* (54.6% of participants), *Fear for baby’s health/life* (49.9%), *High intensity of pain/physical discomfort* (47.4%), and *Communication/explanation* (43.7%). An overview of the answers is shown in Table 2 in descending order of frequency and extended with stratification by parity. This stratification shows that primiparous participants chose *High intensity of pain/physical discomfort*, *Long duration of delivery*, and *Discrepancy between expectations and reality* more often and *Fear for own health/life*, *A bad outcome*, and *Delivery went too fast* less often than multiparous participants.

**Table 2:** Women’s attributions of the traumatic birth experience in descending order of frequency and stratified by parity. Participants could choose multiple answers; there was no maximum number of answers

Answer given	*n*	%	Primiparous	Multiparous	*p* value
Lack and/or loss of control	1196	54.6%	961 (55.3%)	226 (51.2%)	0.13
Fear for baby’s health/life	1093	49.9%	846 (48.7%)	235 (53.3%)	0.09
High intensity of pain/physical discomfort	1039	47.4%	850 (48.9%)	184 (41.7%)	0.01 *
Communication/explanation	957	43.7%	773 (44.5%)	178 (40.4%)	0.12
Long duration of delivery	830	37.9%	746 (42.9%)	78 (17.7%)	0.00 *
Lack of emotional and/or practical support from caregivers	781	35.6%	620 (35.7%)	154 (34.9%)	0.76
A certain action/intervention was done	758	34.6%	608 (35.0%)	145 (32.9%)	0.40
Discrepancy of expectations	751	34.3%	617 (35.5%)	133 (30.2%)	0.03 *
(Lack of) autonomy/involvement in decision-making process	664	30.3%	513 (29.5%)	146 (33.1%)	0.15
Fear for own health/life	633	28.9%	476 (27.4%)	155 (35.1%)	0.00 *
Respect/taken seriously/way they were treated	487	22.2%	375 (21.6%)	106 (24.0%)	0.27
Bad outcome (impactful maternal/infant complications)	444	20.3%	334 (19.2%)	108 (24.5%)	0.01 *
A certain intervention was not done, while the woman would have wanted it to be	382	17.4%	293 (16.9%)	87 (19.7%)	0.16
Lack of emotional support from partner	178	8.1%	134 (7.7%)	42 (9.5%)	0.21
Other					
Separated from baby after delivery	36	1.6%	32 (1.8%)	4 (0.9%)	0.17
Delivery went too fast	34	1.6%	21 (1.2%)	13 (2.9%)	0.01 *
Have not experienced the delivery consciously (due to general anesthesia or other medication)	25	1.1%	22 (1.3%)	3 (0.7%)	0.30
Other reasons	93	4.2%	34 (4.9%)	7 (3.9%)	0.57

^*^Significant at *p* ≤ 0.05

In the category “Other,” many answers fit into three extra topics: *Separated from baby after delivery*, *Delivery went too fast*, and *Have not experienced the delivery consciously* (*due to general anesthesia or other medication*).

Of the 51 participants whose baby had died, 62.7% appointed *A bad outcome* as one of the causes of their traumatic experience. The remaining 37.3% mainly reported *Lack and/or loss of control*, *Communication/explanation*, *Respect/taken seriously/way they were treated*, and *Lack of emotional and/or practical support from caregivers*.

The answer *Discrepancy between expectations and reality* was chosen significantly more often as cause of trauma when the preparation methods *Hypnobirthing* and/or *Reading in books or on the internet* were used, than when these preparation methods were not used (51.7 vs. 33.8%, *p* = 0.004, and 36.4 vs. 31.1%, *p* = 0.010). For other preparation methods, there were no significant differences in how often *Discrepancy between expectations and reality* was perceived as cause of trauma.

### Improvement in caregiver management

Participants were asked what their caregiver could have done to prevent the traumatic birth experience. A minority of 12.4% indicated that the caregiver could have done *nothing* to prevent the trauma. *Communicate/explain* (39.1%) and *Listen to me* (*more*) (36.1%) were the most frequently chosen answers, followed by *Support me* (*more/better*) *emotionally/practically* (29.8%), as shown in Table 3. Examples of lack of emotional or practical support given by women in the free text fields included not being taken seriously in their perception of the speed of labor progression, being left alone during labor, no continuity of care, and a midwife or gynecologist who was too busy to spend time with them. The answers most often ranked as the most important in this category were *Listen to me* (*more*) (20.6%) and *Communicate/explain* (19.7%). Stratification by parity showed that primiparous women listed *Discuss expectations/birth plan*, *Communicate/explain*, and *Do certain actions/interventions later/not at all* significantly more often than multiparous women. Multiparous women chose the options *Nothing* and *Listen to me* (*more*) significantly more often than primiparous women. Examples of commentaries of women in reference to the answer *Discuss expectations/birth plan* were that their birth plan was not taken seriously, that they had not been realistically informed about the likelihood of certain interventions or outcomes, and that their antenatal course downplayed the actual pain involved (“painting a rosy picture”).

**Table 3:** What women believe caregivers could have done to prevent the traumatic birth experience in descending order of frequency and stratified by parity. Participants could choose and rank multiple answers, with a maximum of three

Answer given	*n*	%	Primiparous	Multiparous	*p* value
Communicate/explain	718	39.1%	587 (40.2%)	126 (33.2%)	0.01 *
Listen to me (more)	678	36.9%	515 (35.3%)	160 (42.2%)	0.01 *
Support me (more/better) emotionally/practically	547	29.8%	445 (30.5%)	97 (25.6%)	0.06
Do certain actions/interventions sooner	454	24.7%	367 (25.1%)	86 (22.7%)	0.33
Discuss expectations/birth plan	311	16.9%	271 (18.6%)	39 (10.3%)	0.00 *
Do certain actions/interventions later/not at all	286	15.6%	242 (16.6%)	43 (11.3%)	0.01 *
Don’t do anything without my permission	252	13.7%	207 (14.2%)	44 (11.6%)	0.19
Nothing	228	12.4%	164 (11.2%)	61 (16.1%)	0.01 *
Remain calm	228	12.4%	175 (12.0%)	53 (14.0%)	0.29
Other	54	2.9%	22 (3.2%)	7 (3.9%)	0.63

^*^Significant at *p* ≤ 0.05

### Improvement in self-management

The most frequently chosen answer to the question what participants wanted to have done themselves to prevent the trauma or decrease its impact, was *Nothing* (37.0%) (Table 4). *Ask for certain actions/interventions* (26.9%) and *Refuse certain actions/interventions* (16.5%) were also mentioned frequently. Analysis of the ranking in importance of the given answers identified the same top three. Some examples of actions/interventions mentioned in the free text fields were cesarean section, pain relief, vaginal examinations, and operative vaginal delivery. Stratification by parity showed that primiparous women chose *Be* (*better*) *prepared*, *Make a* (*better*) *birth plan*, and *Refuse certain actions/interventions* more frequently and *Nothing* less frequently than multiparous women. After analyzing themes among frequently mentioned answers within the option *Other*, the categories *Be assertive/express myself/remain in charge* and *Make different choices in caregiver* were added.

**Table 4:** What women wished they had done themselves to prevent the traumatic childbirth in descending order of frequency and stratified by parity. Participants could choose and rank multiple answers, with a maximum of three

Answer given	*n*	%	Primiparous	Multiparous	*p* value
Nothing	677	37.0%	501 (34.7%)	170 (45.6%)	0.00 *
Ask for certain actions/interventions	491	26.9%	400 (27.7%)	89 (23.9%)	0.14
Refuse certain actions/interventions	302	16.5%	252 (17.4%)	49 (13.1%)	0.05 *
Be (better) prepared	293	16.0%	254 (17.6%)	38 (10.2%)	0.00 *
Remain (more) calm/accept	266	14.6%	214 (14.8%)	50 (13.4%)	0.49
Make a (better) birth plan	243	13.3%	211 (14.6%)	32 (8.6%)	0.00 *
Ask support from partner	168	9.2%	142 (9.8%)	26 (7.0%)	0.09
Other					
Be assertive/express myself/remain in charge	91	5.0%	71 (4.9%)	19 (5.1%)	0.89
Make different choices in caregiver	62	3.4%	41 (2.8%)	21 (5.6%)	0.01 *
Other answers	55	3.0%	27 (3.9%)	9 (5.1%)	0.50

^*^Significant at *p* ≤ 0.05

### Other results

After the traumatic childbirth experience, 48.1% of women had a postpartum check-up with the caregiver who attended the delivery, 36.9% with another caregiver, and 15.0% did not have a check-up at all. Of those who did not have a check-up, 4 out of 5 indicated they were not invited and 1 out of 5 chose not to go. Of the women who did have a check-up, 42.0% were asked by the caregiver how they had experienced the delivery and 31.6% brought up the subject themselves. In the remaining 26.4%, the experience of the delivery was not discussed. When the traumatic experience was mentioned by the woman herself, 23% of the caregivers did nothing with this information and some participants added in the free text field that they felt their experience was downplayed (2.3%). According to participants, caregivers might have helped them better if they would have evaluated the experience more thoroughly (62.0%) or if they would have referred them for treatment of the trauma (28.7%).

Almost half of the participants (41.0%) considered filing a complaint against their caregiver, and 7.2% actually did. Of those participants who had a postpartum check-up with the same caregiver who assisted in their delivery, 39.5% considered filing a complaint versus 54.1% who had the check-up with a different caregiver. This difference was significant (*p* = <0.001).

Within the group of participants who had a postpartum check-up without discussing the traumatic experience, 21.3% reported that this was due to the check-up being too soon after the delivery. They explained they could not talk about it yet or they did not yet realize that it was a trauma.

Finally, outcome was compared for level of care during delivery. Participants were stratified into one of three categories: those who received only primary (midwife-led) care (A), those who started their delivery in primary care but were transferred to secondary (obstetrician-led) care during the delivery or immediately postpartum (B), and those who started their delivery in secondary care (C). Concerning perceived cause of the trauma, eight answers showed significant differences between the groups: *Communication/explanation* (A = 32.0%, B = 44.6%, C = 44.3%; AvsB *p* = 0.02; AvsC *p* = 0.02; BvsC *p* = 0.99) and *A certain intervention was done* (A = 23.0%, B = 35.4%, C = 35.7%; AvsB *p* = 0.01; AvsC *p* = 0.01; BvsC *p* = 0.99) were chosen significantly less often in the primary care group (A) than in the other two groups. Women who were transferred during labor (B) reported *A long duration of delivery* (A = 22.1%, B = 48.7%, C = 30.8%; AvsB *p* = 0.00; AvsC *p* = 0.08; BvsC *p* = 0.00) significantly more often than those who received solely primary or secondary care. The secondary care receivers (C) reported *Fear for own health/life* (A = 18.0%, B = 25.4%, C = 33.1%; AvsB *p* = 0.13; AvsC *p* = 0.00; BvsC *p* = 0.00) significantly more often and *High intensity of pain/physical discomfort* (A = 59.0%, B = 49.6%, C = 44.4%; AvsB *p* = 0.12; AvsC *p* = 0.01; BvsC *p* = 0.05) significantly less often than the other two groups. *Fear for baby’s health/life* (A = 35.2%, B = 47.0%, C = 54.1%; AvsB *p* = 0.03; AvsC *p* = 0.00; BvsC *p* = 0.00), *the Delivery went too fast* (A = 7.0%, B = 0.0%, C = 2.0%; AvsB *p* = 0.01; AvsC *p* = 0.04; BvsC *p* = 0.01), and *A bad outcome* (A = 9.0%, B = 18.4%, C = 22.8%; AvsB *p* = 0.00; AvsC *p* = 0.00; BvsC *p* = 0.04) significantly differed between all three groups separately.

Regarding advice to caregivers in order to prevent traumatic delivery experiences, participants who received solely primary care (A) answered *Communicate/explain* significantly less often than those who received solely secondary care (A = 27.6%, B = 38.9%, C = 40.7%; AvsB *p* = 0.054; AvsC *p* = 0.02; BvsC *p* = 0.74). With respect to what participants could have done to prevent the traumatic experience, two significant differences were found between participants who received solely secondary care (C) and those who were referred during labor (B): referred participants chose *Remain calm/accept* (A = 14.0%, B = 17.1%, C = 12.8%; AvsB *p* = 0.77; AvsC *p* = 0.90; BvsC *p* = 0.04) more often and less often reported *Nothing* (A = 33.0%, B = 33.2%, C = 40.3%; AvsB *p* = 1.00; AvsC *p* = 0.32; BvsC *p* = 0.01).

## Discussion

### Main findings

Women attribute the cause of their traumatic birth experience primarily to lack and/or loss of control and issues of communication and practical/emotional support. They believe that in many cases their trauma could have been reduced or prevented by better communication and support by their caregiver or if they themselves had asked for more or fewer interventions.

### Strengths and limitations

The design of this study created the opportunity to quantify the opinions of a much larger sample than has ever been reported in previous qualitative studies. The answer options to the most important questions were based on themes and risk factors identified in previous studies, with the extra option “Other” and room for explanation, which ensured no major themes were missed. Disseminating the questionnaire online through social media proved an efficient way to reach many women with a traumatic birth experience. In January 2016, 85% of the Dutch population within the age category 20–39 years old used Facebook (Van der Veer et al. 2016). More than 95% of women giving birth in the Netherlands, 175.181 in 2014 (CBS 2016), fall within this age category (Brouwers 2014).

Limitations of the study include the inability to generalize findings to all women with a traumatic birth experience due to self-selection of the participants. Certain groups of women may have responded in disproportionate numbers to the recruitment posts. For instance, women with strong convictions about mismanagement of their labor, as well as women who had sought psychological help, could have been more attracted to fill out the survey than women who had found a way to successfully process their experience or who felt safe and well-supported irrespective of an adverse outcome. In addition, we cannot rule out the possibility that some women who experienced physical trauma during their delivery misunderstood the invitation and filled out the questionnaire, when in fact they did not experience any emotional trauma. However, in the Netherlands, the word trauma in lay terms is generally understood to mean psychological trauma. Therefore, we believe we can safely assume most participants interpreted our invitation correctly. Also, more than half of the participants report on a delivery that occurred more than 2 years ago, making recall bias a distinct possibility. The fact that the study took place in the Netherlands, with its unique obstetrical model, may impact on its generalizability. However, many findings are in accordance with previous (qualitative) studies done elsewhere, including need for better emotional support and sense of control. Non-Dutch-speaking women were excluded, leaving out important groups such as immigrants and functional illiterates, who potentially carry higher risks of traumatic birth experiences due to communication difficulties.

### Discussion of the main findings

*Lack and/or loss of control* was most often perceived as a major cause of trauma. This is in line with some previous studies where lack and/or loss of control was identified as a risk factor for PTSD and for experiencing birth as traumatic (Grekin and O’Hara 2014; Ayers et al. 2016; Soet et al. 2003; O’Donovan et al. 2014). This finding was strengthened by 37% of participants reporting that there was nothing they could have done differently, often adding the remark that the situation was not their fault. Also, these results further support the literature concerning the importance of interactions with caregivers (concerning communication, explanation, listening, emotional and practical support) (Grekin and O’Hara 2014; Ayers et al. 2016; Elmir et al. 2010; Harris and Ayers 2012). When situations are thoroughly and clearly explained, fear might decrease, especially for those women who explained their fear was due to not knowing what was happening and why (Vandevusse 1999), which was also an explanation various participants gave to lack or loss of control. Providing information seems important not only during labor itself, but also during pregnancy, as is shown by the number of women who listed *Discuss expectations/birth plan* as a point of improvement for their caregiver. Pain might also decrease with good practical and emotional support from caregivers, as concluded by a Cochrane review finding that continuous support during labor decreases the risk of receiving analgesia and is associated with fewer negative birth experiences (Hodnett et al. 2013).

Another interesting finding was that *preparation for birth with hypnobirthing* was strongly associated with a discrepancy between expectations of the delivery and the reality as cause of the trauma. This raised the question whether this particular approach to birthing adequately prepares women for the reality of labor. However, one could also hypothesize that choice of birthing class could be influenced by expectations or even personality, so this finding would need to be re-examined in a randomized setting. The effects of hypnobirthing, where the woman and her partner are taught self-hypnosis during labor and are told that childbirth does not have to be painful, on delivery and patient satisfaction have not yet been studied in depth (Cyna et al. 2013; Finlayson et al. 2015).

### Interpretation

In previous Dutch studies, referral from primary to secondary care during labor has already been linked to experiencing loss of control (Geerts et al. 2014) and was found to be associated with both a negative birth experience 10 days postpartum and negative recall after 3 years (Rijnders et al. 2008; Kleiverda et al. 1991). In line with this finding, the present study comprises significantly more referred women (43%) than the general population (23%). Our sample also contained significantly fewer exclusively primary care receivers (6%) than the general population (27%), which fits with previous research (Stramrood et al. 2011). There are two likely explanations for this. Firstly, complications and interventions are associated with traumatic delivery experiences and PTSD (Grekin and O’Hara 2014; Ayers et al. 2016; Soet et al. 2003; Stramrood et al. 2011), and many interventions are not possible in primary care (e.g., cesarean section, instrumental delivery, epidural analgesia, induction of labor). Lower rates of trauma in primary care are the logical consequence. Secondly, when women are referred, they will usually encounter a new and unfamiliar team of obstetric care providers, which could influence the quality of patient–provider interaction.

Studies concerning risk factors for PTSD and traumatic or negative birth experiences consistently found operative births (operative vaginal deliveries and cesarean sections) to be a risk factor (Ayers et al. 2016; Soet et al. 2003; Stramrood et al. 2011; Rijnders et al. 2008). Interestingly, while the high prevalence of operative births among our participants suggests the same association, the interventions themselves—being ranked seventh—were not among the most frequently reported causes of trauma. Rather, the traumatic nature of operative births might be linked to the interactions around it, such as the indication for the intervention or the procedure itself not being explained, the woman being insufficiently supported or inadequately prepared for the realities of childbirth. The idea of “interactions rather than interventions” is further supported by the answers from a considerable proportion of the women who had lost their baby. More than one in three participants whose baby died did not report this as a cause of the trauma. Instead, they reported lack and/or loss of control and shortcomings in the interaction with caregivers (communication, respect, and support). This is in line with a recent study among Australian midwives, which found that they too showed stronger reactions to the trauma of disrespectful interpersonal interactions between women and caregivers than to physical trauma or even death (Leinweber et al. 2017).

Expectations appeared to be an important issue for primiparous participants in our study. Caregivers should discuss realistic expectations of delivery during pregnancy and pay sufficient attention to preparation and birth plans. This study also demonstrates that women who have experienced their birth as traumatic do not always receive a postpartum check-up with the caregiver who was present during the birth. This may contribute to underdiagnosis and undertreatment and is a missed opportunity for reviewing the course and experience of giving birth with the caregiver who was present. We recommend that every woman should be offered a postpartum visit with the caregiver who assisted her during their delivery.

## Conclusion

The most important items identified by 2192 women with a traumatic birth experience were lack and/or loss of control and interaction with caregivers (concerning communication/explanation, listening, emotional and practical support). Interaction around interventions seemed to be more important than the interventions themselves, which is crucial information for obstetric care providers to be aware of. Referral from primary to secondary care occurred more often in this group than average in the Dutch birth registry. There is a definite need for attention to and improvement of communication and interaction between patient and caregiver, not only during antenatal care and labor but also during postpartum follow-up.

The findings from this study should form a basis for future research and policy aimed at reducing and preventing traumatic delivery experiences. Further research is needed regarding optimal ways (information provided and courses offered) to prepare women for the reality of birth.

Future studies on the effects of continuity of care(give)r should pay special attention to the experiences and opinions of women who experienced referral during childbirth.
